# Whole transcriptome sequencing reveals HOXD11-AGAP3, a novel fusion transcript in the Indian acute leukemia cohort

**DOI:** 10.3389/fgene.2023.1100587

**Published:** 2023-04-11

**Authors:** Sagar Sanjiv Desai, Febina Ravindran, Amey Panchal, Nishit Ojha, Sachin Jadhav, Bibha Choudhary

**Affiliations:** ^1^ Department of Biotechnology and Bioinformatics, Institute of Bioinformatics and Applied Biotechnology, Bangalore, Karnataka, India; ^2^ Graduate Student Registered Under Manipal Academy of Higher Education, Manipal, Karnataka, India; ^3^ Cancer Centre, Healthcare Global Enterprises Ltd., Bangalore, India

**Keywords:** HOXD11_AGAP3, Indian population, transcriptome, AML, immune profile

## Abstract

**Introduction:** Acute leukemia is a heterogeneous disease with distinct genotypes and complex karyotypes leading to abnormal proliferation of hematopoietic cells. According to GLOBOCAN reports, Asia accounts for 48.6% of leukemia cases, and India reports ~10.2% of all leukemia cases worldwide. Previous studies have shown that the genetic landscape of AML in India is significantly different from that in the western population by WES.

**Methods:** We have sequenced and analyzed 9 acute myeloid leukemia (AML) transcriptome samples in the present study. We performed fusion detection in all the samples and categorized the patients based on cytogenetic abnormalities, followed by a differential expression analysis and WGCNA analysis. Finally, Immune profiles were obtained using CIBERSORTx.

**Results:** We found a novel fusion HOXD11-AGAP3 in 3 patients, BCR-ABL1 in 4, and KMT2A-MLLT3 in one patient. Categorizing the patients based on their cytogenetic abnormalities and performing a differential expression analysis, followed by WGCNA analysis, we observed that in the HOXD11-AGAP3 group, correlated co-expression modules were enriched with genes from pathways like Neutrophil degranulation, Innate Immune system, ECM degradation, and GTP hydrolysis. Additionally, we obtained HOXD11-AGAP3-specific overexpression of chemokines CCL28 and DOCK2. Immune profiling using CIBRSORTx revealed differences in the immune profiles across all the samples. We also observed HOXD11-AGAP3-specific elevated expression of lincRNA HOTAIRM1 and its interacting partner HOXA2.

**Discussion:** The findings highlight population-specific HOXD11-AGAP3, a novel cytogenetic abnormality in AML. The fusion led to alterations in immune system represented by CCL28 and DOCK2 over-expression. Interestingly, in AML, CCL28 is known prognostic marker. Additionally, non-coding signatures (HOTAIRM1) were observed specific to the HOXD11-AGAP3 fusion transcript which are known to be implicated in AML.

## 1 Introduction

India ranks third in leukemia incidence rates worldwide, with a median survival rate of 35.5% (Bahl et al., 2015; Philip et al., 2015). A higher incidence of blood cancer is observed in men (9.5%) compared to women (5.5%) (Ahirwar et al., 2018). The median age of onset is 40 years, a significant departure from developed countries (Philip et al., 2015; Udupa et al., 2020). Of the two major classes of leukemias, Myeloid leukemias are more common in India and lymphoid in the west (Ahirwar et al., 2018).

Amongst the 7 subtypes of AML, M2 is the most common subtype of AML in the Indian population (Bhattacharyya et al., 2018; Udupa et al., 2020). Cytogenetic abnormalities, such as a change in chromosome number or chromosomal translocations, are considered hallmark genetic abnormalities of hematopoietic neoplasms (Küppers and Dalla-Favera, 2001; Nambiar et al., 2008; Falini et al., 2010). These chromosomal rearrangements lead to fusion transcripts frequently known to drive cancers. Philadelphia chromosome, t (9; 22): BCR-ABL1 was reported in chronic myeloid leukemia (CML), and is used as diagnostics for CML and also some uncertain cases of acute leukemia, and is associated with the worst prognosis in children and adults (Kang et al., 2016; Sampaio et al., 2021). The fusion transcripts resulting from the translocation lead to abnormal cell proliferation.

Detection of fusion transcripts has become robust after the emergence of NGS technologies like transcriptome sequencing, advanced sequencing platforms, and analysis tools (Kumar et al., 2016). The use of fast and accurate tools like STAR-Fusion, which works on RNA-seq data, has been reported to be among the best performers (Haas et al., 2019).

Targeted exome sequence analysis revealed the HOXD11-AGAP3 fusion gene in pediatric nervous system tumors and gastric tumors (Yuan et al., 2022) (Wu et al., 2019). AGAP3, an NMDA receptor signaling complex component, is suggested to be related to GTP binding activity (Oku and Huganir, 2013). GTP binding proteins have been shown to express in leukemic cell lines and could have a role in the cellular differentiation of megakaryoblasts (Nagata et al., 1995). Moreover, Rac, A GTP-binding protein, is known to play a pivotal role in BCR/ABL driven Leukemic conditions (Skorski et al., 1998).

Newman et al. developed a method to fractionate the immune cells using gene expression, CIBERSORT (Cell type Identification By Estimating Relative Subsets Of known RNA Transcripts) (Newman et al., 2015). It has been utilized in AML patients to predict inferior event-free survival (EFS) and overall survival (OS) and identifying novel prognostic markers (Wang et al., 2021). The relative presence of M0 vs. M2 macrophages predicted the relapse. Apart from the available risk stratification markers, the immune cell scoring system (ICSS) can be used as an independent prognostic predictor (Wang et al., 2021).

Co-expression analysis to understand disease pathogenesis has been widely used (Sundarrajan and Arumugam, 2016). Recent studies have utilized weighted gene co-expression network analysis (WGCNA) to identify co-expressed groups of genes from RNA-seq (Clarke et al., 2013), which is based on higher-order relationships and integrated function of gene modules (Zhao et al., 2010; Kadarmideen and Watson-Haigh, 2012).

There are limited NGS-based AML studies from India. Jain et al. (2020) performed NGS analysis on two AML patients and reported that NGS-guided therapy was better than chemotherapy, which was associated with poor survival (Jain et al., 2020). 34 AML patient samples mutation profiling was performed using WGS, and WES highlighting the differences between the genomic landscape of Indian AML and the published literature, and Patkar et al. used 34 gene panel targeted NGS to detect minimal residual disease (MRD) in AML (Dalal et al., 2019; Patkar et al., 2021).

In this study, we have performed transcriptome sequencing and analysis of 9 Indian patient samples from an HCG hospital in Bangalore. Primarily, we performed fusion detection in all the samples and detected a novel fusion HOXD11-AGAP3 in 3 patients, BCR-ABL1 in 4 patients, and KMT2A-MLLT3 in one patient. We performed differential expression of BCR-ABL1 patients and HOXD11-AGAP3 patients independently. The differentially expressed (DE) genes were used as input for WGCNA analysis, which revealed fusion transcript-specific enriched pathways. We also checked the samples’ immune profiles and found distinct differences between the patients and immune cells correlated with HOXD11-AGAP3 fusion.

## 2 Methods

### 2.1 Subjects for the study

We obtained 9 peripheral blood samples diagnosed with AML at the Healthcare Global Enterprises Ltd., Bengaluru, Karnataka, India. The protocol was approved by the institutional review board of HCG and the Institute of Bioinformatics and Applied Biotechnology. Table 1 lists the clinical details of all the patients. Informed consent was obtained from all the participants.

**TABLE 1:** Patient details and alignment statistics of the 10 Acute Leukemia Samples.

ID	Age	Gender	Type	Number of reads (millions)	Alignment percentage (%)
TL_1	NA	F	AML	120.9	86.23
TL_2	38	M	AML	107.7	93.92
TL_3	50	F	AML	107	93.97
TL_5	45	M	AML	223	87.68
TL_6	55	M	AML	93	93.26
TL_7	51	M	AML	111.8	82.27
TL_8	NA	F	AML	100.8	94.46
TL_9	55	M	AML	108.8	91.88
P3	22	M	AML	159	82.82

### 2.2 Sample collection and culture of PBMCs

The Indian acute leukemia blood samples were collected in anticoagulant EDTA/HEPARIN (3–5 mL each) vacutainers. PBS (equal to that of the sample) was added to the samples, followed by layering the blood on lymphocyte separation media (LSM) (1:2 LSM: blood) along the walls of the tube and centrifuged at 500 RCF for 30 min. The buffy coat was collected and washed 3 times with PBS at 1,000 rpm for 10 min. The washed pellet (cells) was resuspended in 5–10 mL RPMI media (supplemented with 10% FBS) and cultured in T-25 flasks at 37°C with 5% CO2. After the cells reached confluency, they were passaged. The cells were washed with PBS and stored at −80°C in 500 µL of Trizol Reagent.

### 2.3 Total RNA extraction

RNA extraction was carried out using Trizol Reagent (Ambion). 0.5 × 10^6^ cells were resuspended in 500 µL of Trizol, 1/10 vol of 2 M sodium acetate pH4) was added to the tube and mixed, followed by 100 µL of chloroform and mixed vigorously and incubated on ice for 20 min. RNA precipitation was done using an equal volume of isopropanol, and the pellet was washed with 75% ethanol. The pellet was dried and resuspended in DEPC-treated MilliQ water. RNA concentration and purity were checked using Qubit, and its integrity was examined by capillary electrophoresis (Tapestation 2200 Agilent) to ensure RNA integrity number >9 for RNA library preparation.

### 2.4 Illumina Truseq total RNA library preparation and sequencing

For transcriptome sequencing, total RNA libraries were prepared using Illumina TruSeq RNA Library Prep Kit v2. Briefly, the mRNA was isolated from total RNA using oligo-dT beads (NEB; New England Biolabs, Ipswich, Massachusetts, United States). The isolated mRNA was then fragmented to 200-250 bp, followed by double-stranded cDNA synthesis. Adapter ligation was performed after end-repair, and PCR amplification was performed. After constructing the libraries, their concentrations and insert sizes were confirmed using Qubit and Agilent Tapestation, respectively. Library sequencing was outsourced to Medgenome, Bengaluru, Karnataka, India, to obtain 150-bp paired-end reads.

### 2.5 Transcriptome analysis of acute leukemia samples

Raw reads obtained after sequencing were quality-checked using FastQC (https://www.bioinformatics.babraham.ac.uk/projects/fastqc/) and trimmed off sequencing adapters using Trim Galore (https://www.bioinformatics.babraham.ac.uk/projects/trim_galore/). The transcriptome raw data is available on NCBI-SRA (https://www.ncbi.nlm.nih.gov/sra/?term=PRJNA915202). Quality-checked reads were aligned with the standard paired-end alignment options to the human hg38 genome using the STAR aligner (Dobin et al., 2013). The sorted alignment files were indexed using SAMTools (Li et al., 2009), and the read counts were generated using the multiBamCov utility of the BEDTools suite (Quinlan and Hall, 2010). Normalized expression values were calculated from the read counts (TPM-transcripts per million) using in-house python scripts. Fusion genes were identified in the samples using the STAR-Fusion aligner and the FusionInspector module incorporated to validate the identified fusion transcripts (Haas et al., 2019; Haas et al., 2022). Clusters were obtained from PCA analysis using the online ClustViz tool with normalized TPM values (Metsalu and Vilo, 2015). AGAP3 expression values were extracted for the Indian cohort, and a boxplot was generated using the boxplot package in R. The online database GEPIA2 (Tang et al., 2019) was used to obtain a boxplot of AGAP3 expression values in LAML vs. normal samples from the TCGA dataset, along with the Kaplan-Meier survival plot between high-expression and low-expression AML groups.

### 2.6 Immune cell fractions across fusion types

To determine the relative immune cell fractions in all the samples using their transcriptome profiles, the samples were compared against the two whole blood control samples. The immune cell fractions were subsequently compared between samples using the online tool CIBERSORTx. CIBERSORTx has a predetermined set of signature genes defined for every immune cell type and their standard expression values known as the Lm22 matrix. Uploading your transcriptome data into the software, it is compared to the Lm22 matrix, and the percentage of immune cell fraction in every sample is determined.

### 2.7 Differential expression and co-expression networks

The samples were divided into BCR-ABL1 samples and HOXD11-AGAP3 samples. Differential expression was performed for both categories with the two whole blood samples as control using the Bioconductor package DESeq2 in R (Love et al., 2014). Genes were considered upregulated with a log2FC cutoff of +1 and downregulated with −1. The entire transcriptome profile and the differentially expressed genes were subjected to a gene co-expression network analysis using the WGCNA package in R. The most significantly correlated modules were subjected to pathway analysis using the REACTOME database.

### 2.8 Statistical analysis and plots

Samples were normalized using the voom method, and boxplots were generated using the raw and normalized libraries in R. PCA plot was generated online using the ClustVis tool. The fusion transcripts were visualized using the IGV genome browser. The stacked bar chart for immune profiles and the pathway charts were plotted using Microsoft excel. Results were considered to be significant with a *p*-value <0.05. GEPIA2 shows the difference in survival plots to be significant using the log-rank test and the difference in expression to be significant using the Student’s t-test. The co-expression modules were correlated with the traits in WGCNA *via* the Pearson correlation coefficient.

## 3 Results

### 3.1 Patient details and clinical characteristics

We obtained 9 AML samples from the HCG hospital with a median diagnosis age of 50 years, with 3 of them being females and 6 males. Transcriptome library preparation and subsequent RNA-seq analysis was performed for all 9 transcriptome samples. The depth covered per sample was approximately 100X with an average of 116 million reads of length 151 each. After the quality check, an average of 87% alignment was obtained for all the samples (Table 1).

### 3.2 PCA analysis revealed distinct clusters obtained using the normalized transcriptome profiles

**FIGURE 1:**
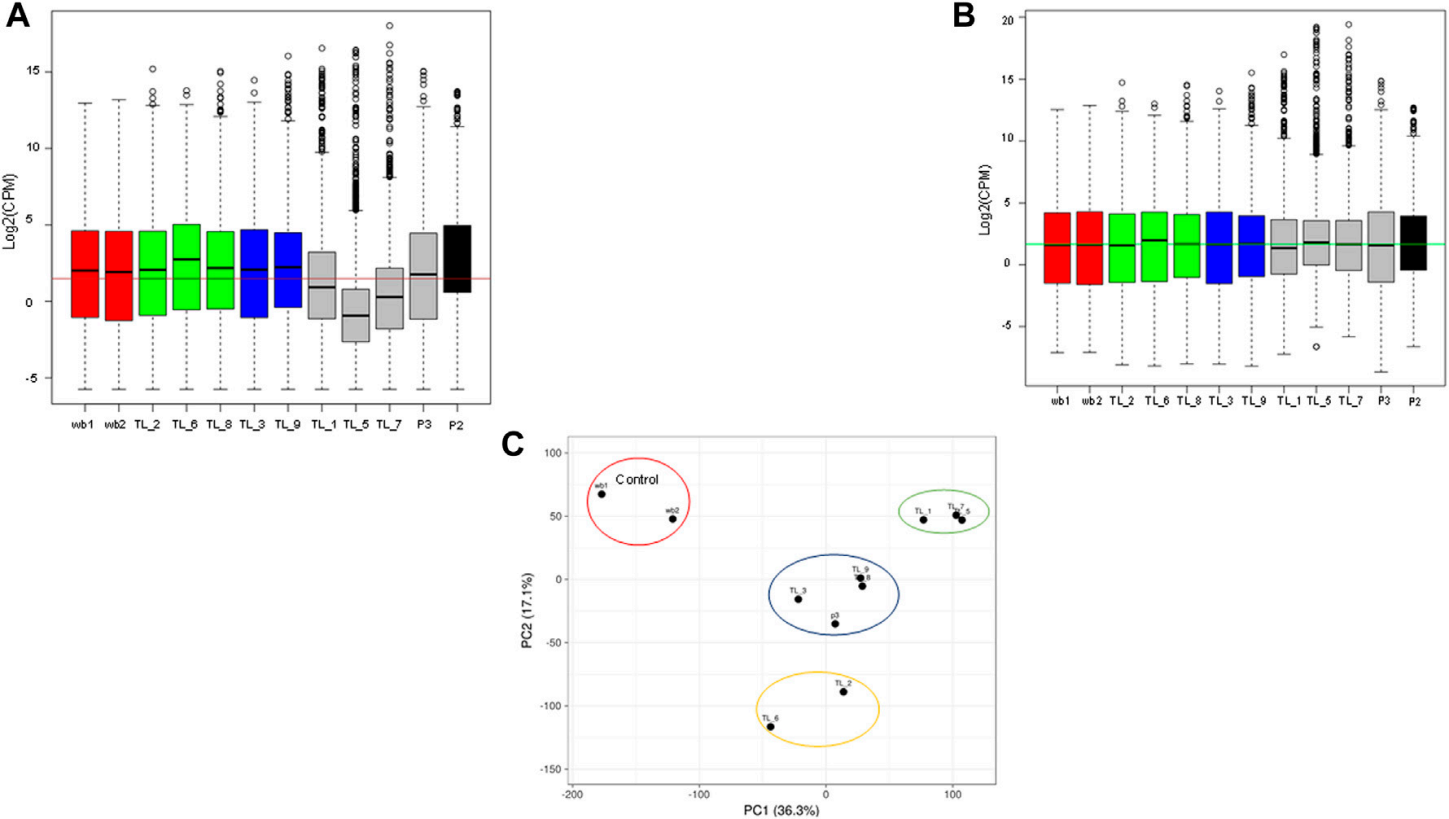
**(A)** Boxplot showing the raw library counts of all the samples involved in the analysis. **(B)** Boxplot of voom normalized libraries of all the samples. **(C)** PCA plot between whole blood control samples and AML samples showing three different clusters within the AML samples.

To normalize sequencing variations between the samples, quantile normalization of the raw reads was performed using the voom normalization method (Figures 1A, B). Further, we performed PCA analysis, and the plot was generated using the online ClustViz tool. To obtain clusters, we got two normal whole blood samples as controls (SRR17278683 and SRR17278702) from NCBI SRA (Brennan et al., 2022). All the AML samples clustered together and away from the 2 control samples, indicating similarity among AML samples (Figure 1C). Further, on observing only the AML samples from the PCA analysis, three clusters were obtained (TL_1, TL_5, and TL_7), (TL_9, TL_8, TL_3, P3), and (TL_2 and TL_6), indicating differences among the three clustered groups in gene expression (Figure 1C). To account for the cluster-wise differences in the samples, we performed fusion transcript analysis to check whether the samples from different clusters harbored different fusion transcripts.

### 3.3 Transcriptome analysis reveals novel fusion gene HOXD11-AGAP3

**FIGURE 2:**
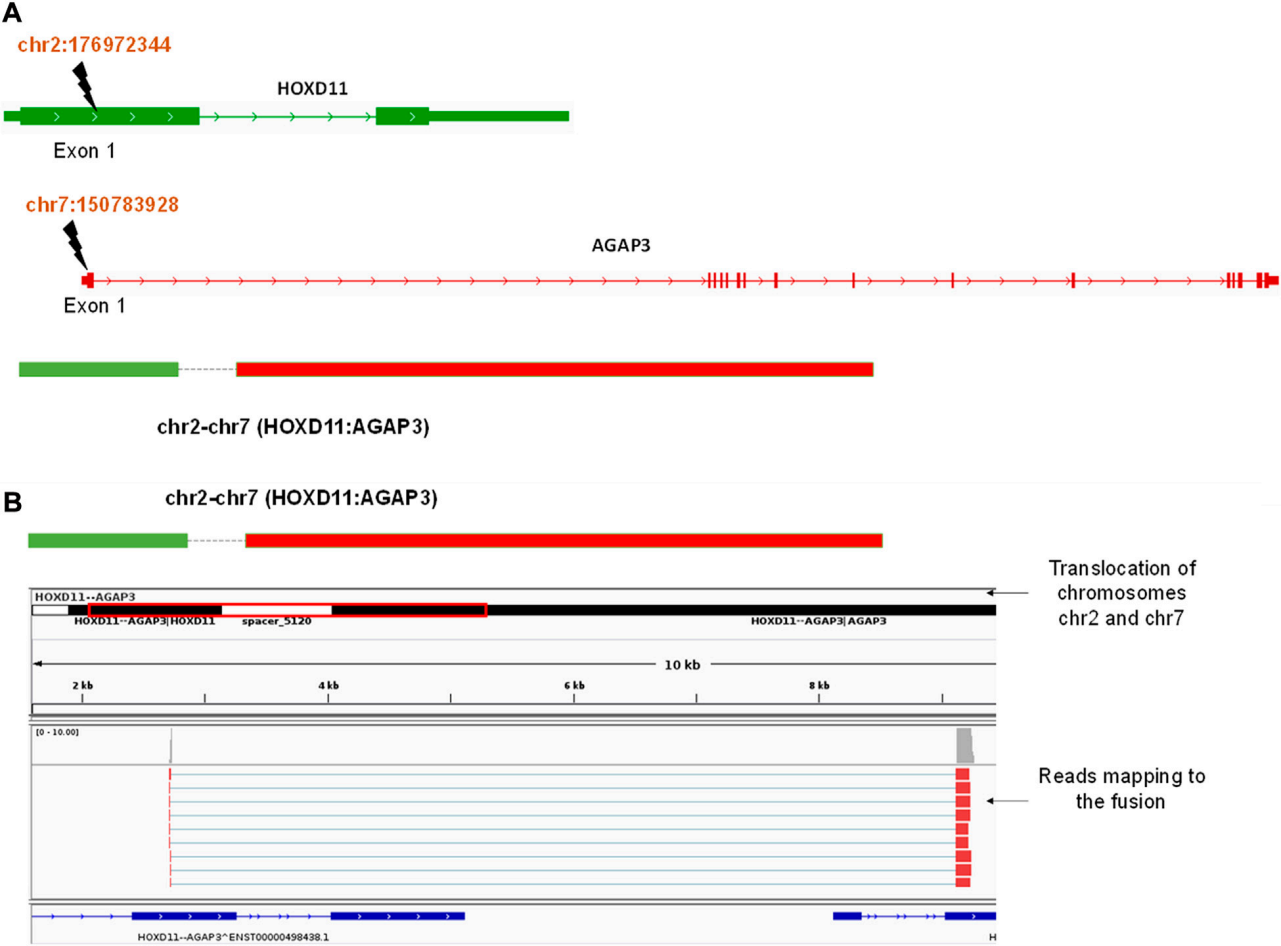
**(A)** A diagrammatic representation of HOXD11-AGAP3 fusion from chr2 and chr7 respectively at the reported locations of Exon 1 in each gene. **(B)** An IGV snapshot showing the HOXD11-AGAP3 fusion transcript with reads from the alignment file mapping to the junction.

We obtained three different fusions by performing fusion detection using the STAR-Fusion tool. The first was BCR-ABL1 fusion in 3 out of 9 samples, TL_2, TL_6, and TL_8 (Supplementary Figure S1, Table 2). TL_2, TL_6, and TL_8 have the same orientation of the BCR-ABL1 fusion with the same breakpoint locations in their respective exons across all three samples. But, TL_8 also has an ABL1-BCR fusion with different exon locations. This could explain the inconsistencies in the transcriptome profiles and the clustering of TL_2 and TL_6 away from TL_8. Secondly, we observed a novel fusion transcript formed between Exon 1 of HOXD11 on chr2 at position 176972344 and Exon1 of AGAP3 on chr7 at position 150783928, resulting in a chr2:chr7/HOXD11-AGAP3 fusion in samples TL-3, TL-6, and TL-9 (Figure 2A; Table 2). Additionally, TL-9 showed a KMT2A-MLLT3 fusion. To further validate the presence of these fusion transcripts, we ran the FusionInspector tool as a part of the STAR-Fusion output. We observed that all three fusions, BCR-ABL1, KMT2A-MLLT3, and HOXD11-AGAP3, had a significant number of reads spanning the respective junctions (Figure 2B, Supplementary Table S1). Interestingly, it was also seen that for all the fusion transcripts detected, the breakpoints in the participating genes occurred in the exonic regions, except HOXD11-AGAP3, which was due to a non-reference splice site involving intron 1 of HOXD11. The fusion sequences with the exact junctions have been depicted in the Supplementary Document.

**TABLE 2:** Fusion genes detected in Indian Acute Leukemia Samples.

Patient ID	Fusion gene detected
TL-1	--
TL-2	BCR; ABL1
TL-3	HOXD11; AGAP3
TL-5	--
TL-6	BCR; ABL1, HOXD11; AGAP3
TL-7	--
TL-8	BCR; ABL1
TL-9	KMT2A; MLLT3, HOXD11; AGAP3
P3	--

We performed ORF analysis on the fusion transcript harboring roughly 3 Kb long intron to check whether a functional AGAP3 protein or a non-functional truncated protein would result. Using ORF finder, we could observe that the HOXD11 homeobox domain fuses with the AGAP3 arfGAP GTPase domain (Supplementary Figure S2A) and from the fusion transcript, we found the longest ORF to be in the frame and had GTPase domains intact (Supplementary Figure S2B), although due to the presence of N nucleotide, it could not be confirmed whether the full-length protein was made. In the absence of a full-length transcript with no clarity on the truncated transcript or full length, we decided to check for the expression of AGAP3 in AML samples.

**FIGURE 3:**
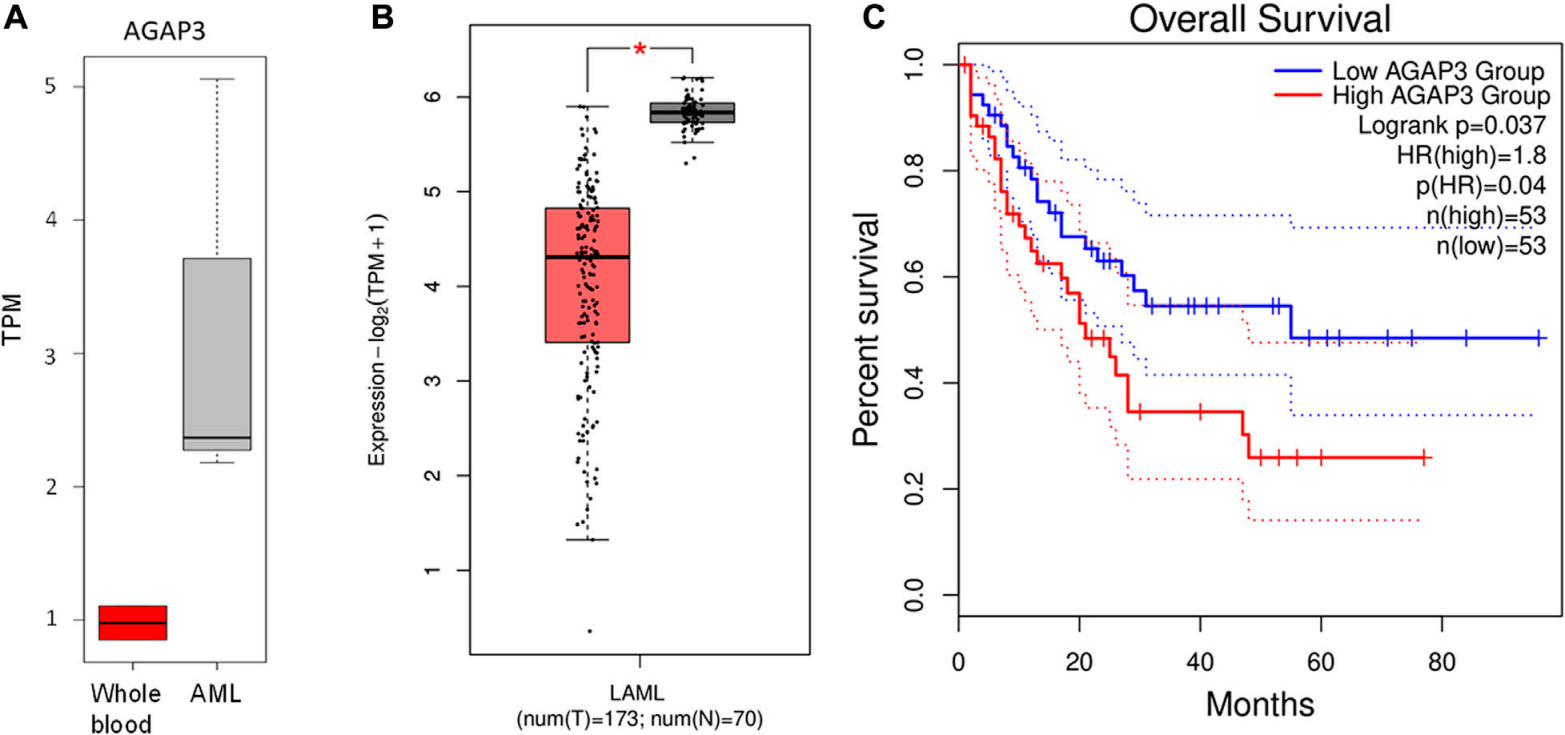
**(A)** Boxplot showing the difference in AGAP3 expression between control and Indian AML samples. **(B)** Boxplot showing a significant difference in AGAP3 expression between the AML and normal samples obtained GEPIA2 database. **(C)** Kaplan-Meier plot showing a significant difference in survival probabilities between low (blue) and high (red) AGAP3 expression groups.

AGAP3 was upregulated in acute leukemia samples compared to the blood control samples (Figure 3A). In comparison, we saw that AGAP3 was downregulated in TCGA AML samples from the GEPIA2 database (Figure 3B), which suggests an Indian population-specific AGAP3 expression pattern. It was also seen that patients with higher AGAP3 expression have a significantly lower survival probability (*p*-value = 0.037) (Figure 3C) in the Caucasian population. We also obtained the interacting partners of AGAP3 from the STRING database, of which TTYH3, which has a significant effect on survival probabilities in AML samples (*p*-value = 0.03), was significantly upregulated in acute leukemia samples (Supplementary Figure S3). We extracted the expression profiles of AGAP3 interacting partners and HOXD11 interacting partners. It was seen that out of the 64 transcription factors binding to AGAP3, ARNT and EP300 were upregulated in TL_3, TL_6, and TL_9 and TTYH3 an interacting partner was the only one upregulated in all three samples with fusion. Amongst the 84 activators of HOXD11, ZNF223 and ARNT were expressed in the samples with HOXD11-AGAP3 fusion proteins.

It was interesting to note that HOXD11:AGAP3 signatures were evident in the expression profiles of lncRNAs associated with genes in their vicinity and the transcripts ZMYM4, ZMYM4_AS1, EP300, LIMS1, LMS1_AS1 SENCR, HOXA2, HOTAIRM1, ZBED3, NNT, NNT_AS1, PWAK1, MACC1, MACC1_AS1, showed a HOXD11-AGAP3 fusion sample-specific expression profile (Supplementary Figure S4).

### 3.4 WGCNA reveals hub genes and networks specific to fusion genes

**FIGURE 4:**
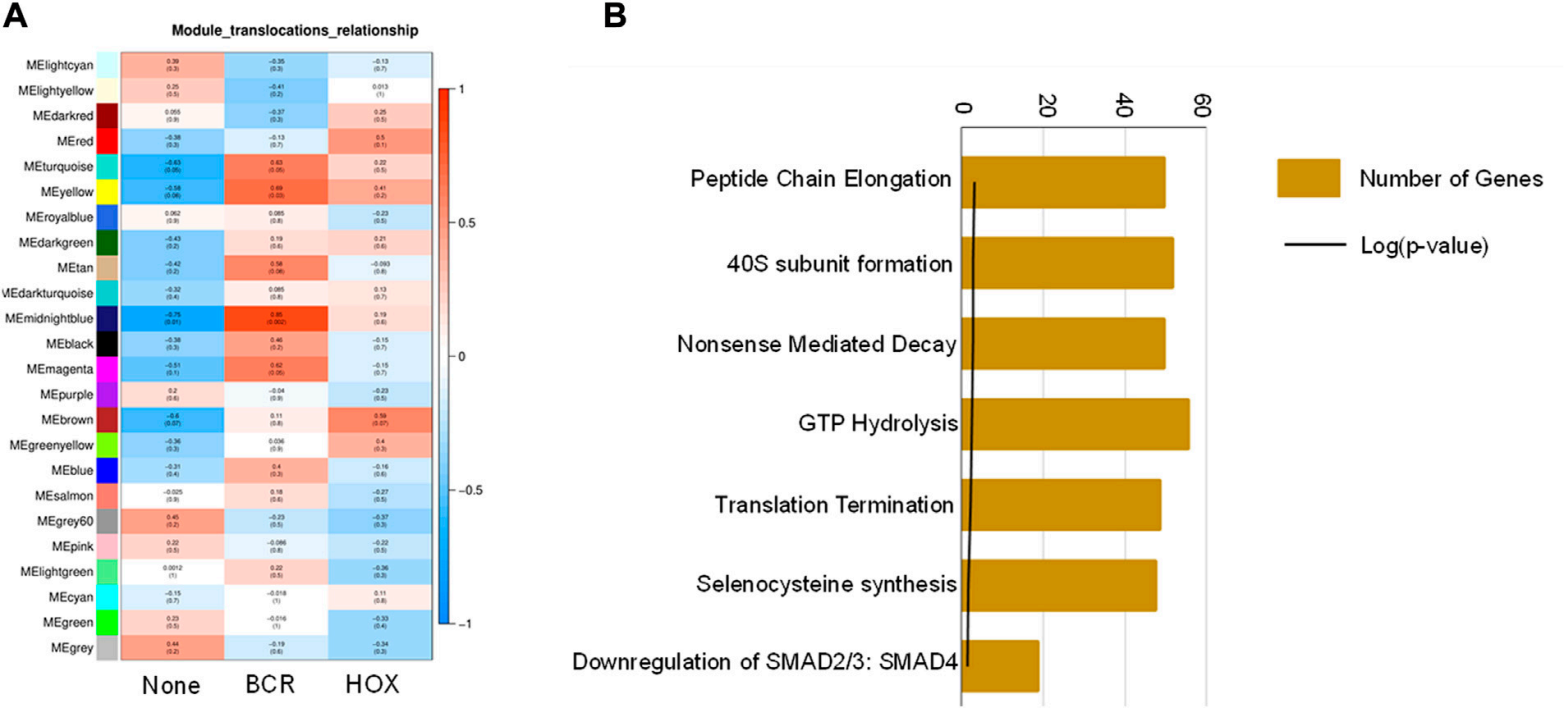
**(A)** Module-trait correlation heatmap using the entire transcriptome dataset with fusion transcript groups as traits, the red color corresponds to positive correlation and the blue color corresponds to negative correlation. **(B)** Bar chart showing significantly enriched pathways obtained using the genes of the “midnightblue” module correlating to the HOX group of samples.

**FIGURE 5:**
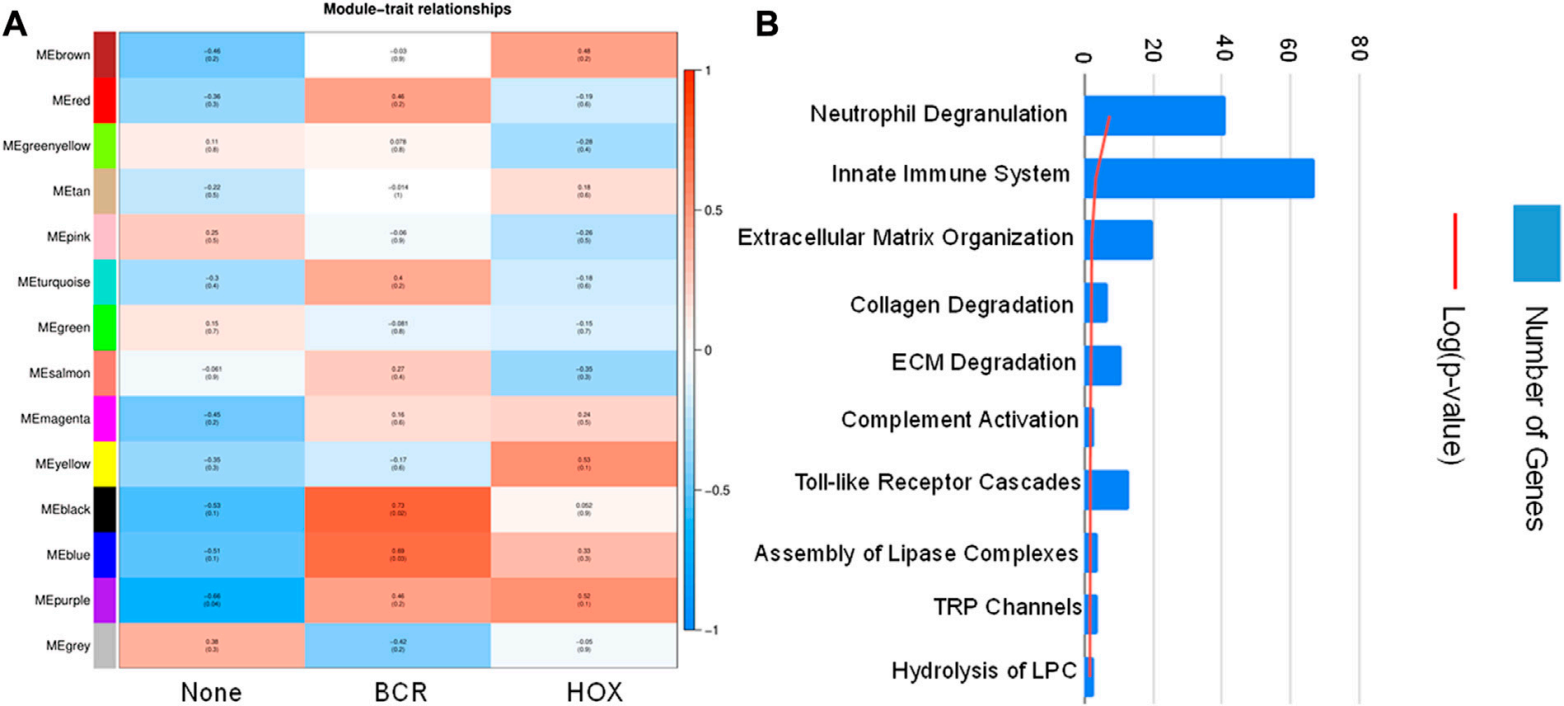
**(A)** Module-trait correlation heatmap using the differentially expressed genes with fusion transcript groups as traits, the red color corresponds to positive correlation and the blue color corresponds to negative correlation. **(B)** Bar chart showing significantly enriched pathways obtained using the genes of the “yellow” module correlating to the HOX group of samples.

To identify co-expression networks specific to the three fusion gene categories (none, BCR and HOX), we performed a WGCNA analysis with TPM values of all the genes with the presence/absence of a fusion transcript (Table 2) as traits. WGCNA starts by constructing a gene co-expression network followed by identifying gene modules based on hierarchical clustering. It correlates these modules to the clinical data/parameters provided by the user and provides correlations values along with the significance of the correlation. The user can then select the most significantly correlated parameter-module pair and proceed with pathway analysis or network analysis of the genes in the module It was seen that the most significantly correlated module for the BCR-ABL1 patients was “midnight blue” (*p*-value = 0.002, Pearson correlation value, r = 0.85) with 91 genes that accounted for transcriptional regulation pathways (Figure 4A).The most significantly correlated module for HOXD11-AGAP3 samples was “brown” (*p*-value = 0.07, r = 0.6), with 4435 genes. The most enriched pathways were peptide chain elongation and termination, 40S ribosomal subunit formation, non-sense mediated decay, GTP hydrolysis, and downregulation of SMAD2/3 and SMAD4 (Figure 4B). To further investigate the effect of different fusion transcripts, we performed differential expression of BCR-ABL1 samples and HOXD11-AGAP3 samples, with whole blood samples as controls. In the case of BCR-ABL1 samples, 1938 genes were differentially expressed (*p*-value <0.05), with 62.34% being upregulated (log2FC > 2) and 37.66% (log2FC < −2) being downregulated. We obtained a total of 3197 differentially expressed genes (*p*-value <0.05, log2FC > 2) in the HOXD11-AGAP3 case, of which 44.13% were upregulated, and 55.87% were downregulated (log2FC < −2). The differentially expressed genes (5135: 1938 from BCR-ABL1 samples +3197 from HOXD11-AGAP3 samples) were fed into another iteration of WGCNA analysis. The module-trait correlation matrix showed that the “black” module is the most significantly correlated with the BCR category (*p*-value = 0.02, r = 0.73), and the “yellow” module shows the best correlation with HOX category (r = 0.53, *p*-value = 0.1) (Figure 5A). On performing pathway analysis, we saw that the black (BCR) module was enriched with gene expression regulation and collagen biosynthesis pathways (Supplementary Figure S5), and the yellow (HOX) module showed neutrophil degranulation, innate immune system, ECM organization, complement activation TLR-cascades, lipase complex assembly, to be the most altered (Figure 5B).

To get a better understanding of the immune system-related effect of HOXD11-AGAP3, we screened for additional signatures and amongst the chemokines, we obtained CCL28 and DOCK2 to be highly expressed in HOXD11-AGAP3 samples as compared to the rest. Additionally, we also observed that difference in CCL28 expression has a significant impact on patient survival (*p* = 0.016) (Supplementary Figure S6).

Since the fusion genes correlated with alterations in the immune system, we profiled immune cells to identify fusion-associated changes in the immune cell fractions.

### 3.5 Immune cell profiling of Indian patient samples

**FIGURE 6:**
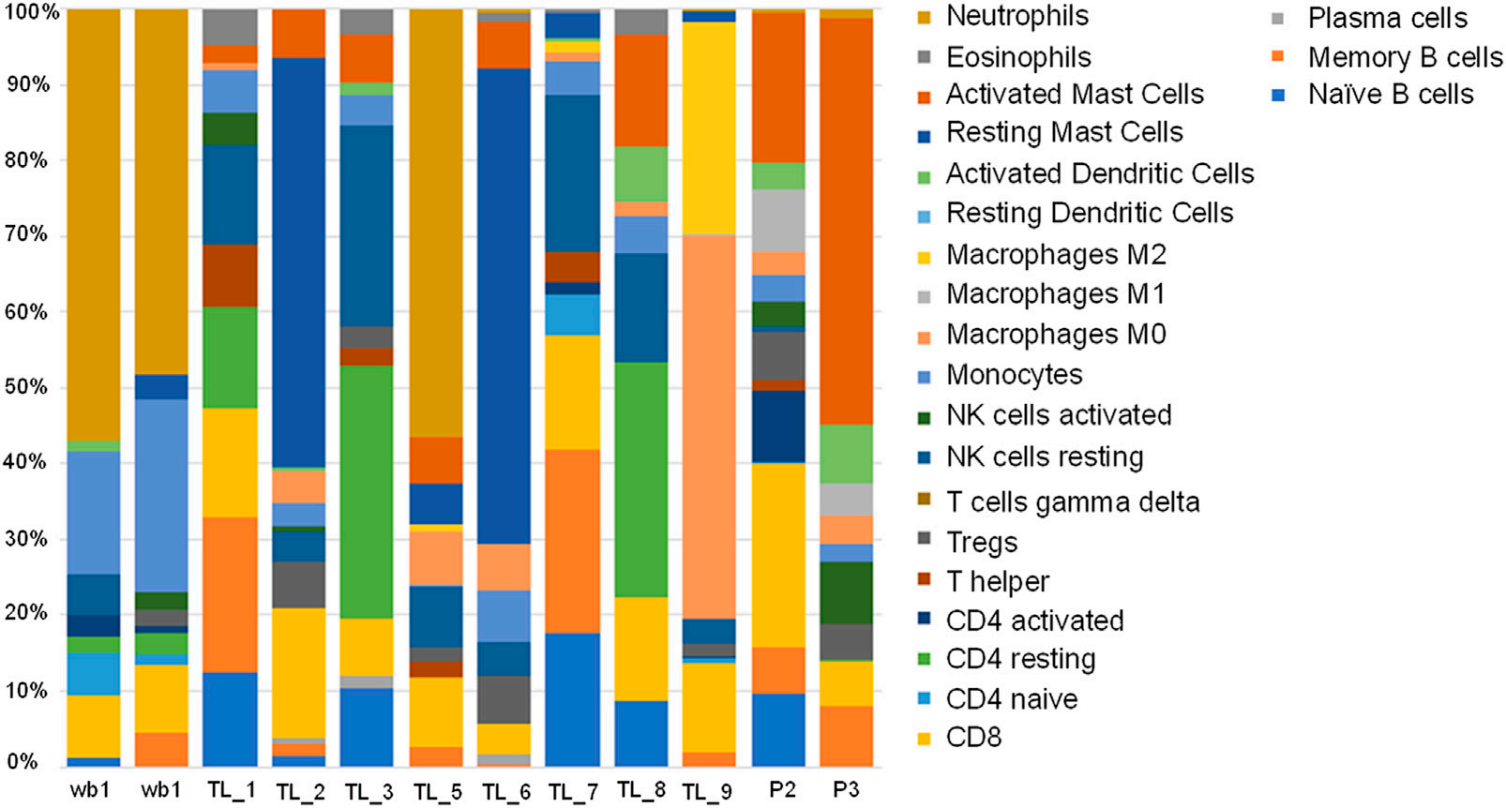
Stacked bar plot depicting 22 immune cell fractions across the control and Acute leukemia samples in terms of percentage.

We evaluated the immune cell profiles with CIBERSORTx. It uses a set of genetic markers for 22 immune cells as reference, against which user provided expression values are compared to determine the percentage of immune cells present in the patient samples being analysed. All the acute leukemia samples had a very different immune profile compared to the whole blood controls. We checked for the immune profile in 3 different groups depending on the fusion transcripts. Among the groups with no fusion transcript (TL_1, TL_5, TL_7, and P3), TL_1 and TL_7 showed a very similar distribution of immune fractions with high mast cell and negligible neutrophil fractions (Figure 6). TL_5 was the only one with a significantly high fraction of neutrophils (45%) compared to the negligible levels in others (Figure 6 13). In the group with BCR-ABL1 fusion (TL_2, TL_6, and TL_8), TL_2 and TL_6 were similar with a higher fraction of Mast cells, Tregs, Macrophage M0, and CD8 cells, while TL_8 had a higher fraction of activated dendritic cells. In the subgroup HOXD11:AGAP3 fusion, all three TL_3, TL_6, and TL_9 was dissimilar in immune fractions, which could be due to the presence/absence of additional fusion in those samples. TL_3, the only sample with just the HOXD11-AGAP3 fusion, had a high frequency of resting CD4 cells and NK cells, followed by CD4 naive and CD8 cells. TL_6’s (that also showed BCR/ABL1 fusion) immune profile was closer to the TL_2 samples than as compared to TL_3 and TL_9. TL_9 showed the highest M0 and M2 macrophage fractions and a relatively high fraction of CD8 cells (Figure 6), indicating patient-specific immune cell fractions. We also observed that there was an exclusive representation of plasma cells in TL_3 and TL_6 samples. TL_3 was also enriched in NK cells, while TL_3 and TL_9 was enriched in CD4 T cells (Figure 6).

## 4 Discussion

One of the main objectives of this study was to identify and characterize karyotypes in the Indian acute leukemia population. Several studies have used transcriptome sequencing data to detect fusion transcripts, but no study has been conducted in India (Lilljebjörn et al., 2013; Stengel et al., 2018; Mata-Rocha et al., 2019). In India, there have been case study reports regarding treatment outcomes of chemotherapy, the genome landscape of two patients, and a study to detect MRD in AML using NGS, but so far, transcriptomics has not been used for any clinical applications (Dalal et al., 2019; Jain et al., 2020; Patkar et al., 2021). Our study reveals patient-specific complex karyotypes, including BCR-ABL1, KMT2A-MLLT3, and a novel fusion transcript HOXD11-AGAP3 in three out of the 10 patients. A differential expression followed by WGCNA analysis resulted in different gene-coexpression networks getting enriched according to the presence of the fusion transcripts.

For the longest time, BCR-ABL1 fusion was believed to be a hallmark of CML patient samples, but recent studies have described a rare category of AML with BCR-ABL translocation (Kang et al., 2016; Neuendorff et al., 2016; Sampaio et al., 2021). Interestingly, we detected BCR-ABL fusion in TL_2, TL_6, and TL_8, which suggests that these samples could have a CML origin with primary blast crisis, which is the case with TL_2, or they could belong to the recently discovered rare category of AML, which are BCR-ABL positive (Neuendorff et al., 2016). In three samples, TL-3, TL-6, and TL-9, we detected a novel fusion gene pair, HOXD11-AGAP3. This fusion gene has been reported in neuroblastoma and gastric tumor cases but not in AML cases (Wu et al., 2019) (*Yuan et al., 2022*). Interestingly, TL_6 also had the BCR-ABL1 fusion transcript and the KMT2A-MLLT3 fusion gene in TL-9. Suggests that TL_6 and TL_9 are complex karyotypes.

Approximately 10%–12% of all AML samples are known to have complex karyotypes, yet they are not very well characterized at the molecular level (Mrózek et al., 2019). AGAP3 is an NMDA receptor signaling complex; its fusion gene is suspected to be involved in GTP-binding and GTPase signaling (Oku and Huganir, 2013; Wu et al., 2019). WGCNA analysis revealed a significant correlation between the HOXD11-AGAP3 fusion and GTP hydrolysis. It has been shown that BCR-ABL activates proteins of the GTP-binding network in the context of leukemogenesis (Skorski et al., 1998). Therefore, the fact that TL_6 has both BCR-ABL1 and HOXD11-AGAP3 could be of great genetic relevance for AML.

Long non-coding RNA in AML has been reviewed thoroughly (Ng et al., 2019). Long non-coding RNA regulates gene expression by binding to the DNA, RNA or protein and directing the localization of the protein. They have been used as diagnostic and prognostic indicators of disease progression, especially cancer. LincRNA is known to regulate cell proliferation and apoptosis of cancer cells (presented in the literature review). One of the lincRNA HOTAIRM1 (present in HOXA gene cluster) overexpression has a well-known impact on prognosis in a myeloid cell differentiation manner in AML (Zhang et al., 2014) (Gao et al., 2020). Interestingly, HOTAIRM1 and HOXA2 expression levels are elevated in HOXD11-AGAP3 samples.

CIBERSORTx analysis showed a distinct pattern of immune cells across the individuals and did not follow fusion gene patterns indicating the fusion products might not influence them. However, mild similarities between the patients were observed, indicating the heterogeneity of AML. TL_5 has a significantly high (45%) neutrophil content which was not observed in any of the samples of the Caucasian population. It has been observed that the neutrophil-to-lymphocyte ratio in the pretreatment group has a better prognosis than NLR high (Zhang et al., 2021). It is possible that the high neutrophil count in the TL_5 sample could be due to an infection and may indicate a bad prognosis. A recent study reported AGAP3 in Alzheimer’s disease correlates with resting and naive CD4 cells, NK cells, CD cells, etc. (Zhao et al., 2022). TL_3, the only sample with just HOXD11-AGAP3, showed high frequencies of the same. WGCNA analysis of the full transcriptome profile enriched the innate immune system and neutrophil degranulation pathways. HOXD11-AGAP3 signatures also included elevated CCL28 and DOCK2 expression. Altered CCL28 expression, which shows a significant effect on survival in AML samples (GEPIA), has been associated with chemokine responsiveness in AML patients. DOCK2, which is expressed only in hematopoietic tissue, is known to be of prognostic value in AML patients (Brenner et al., 2016) (Hu et al., 2019). These facts suggest an immune cell-related role of HOXD11-AGAP3 in AML.

The small sample size of patients in the study is a definite shortcoming, but our results reveal a novel fusion in 3 out of 10 samples, with two being complex Karyotypes. This and other results reveal Indian population-specific signatures for Acute Leukemia that need to be explored and validated further with a larger dataset.

## Data Availability

The data presented in the study are deposited in the NCBI-SRA repository, accession number PRJNA915202 (https://www.ncbi.nlm.nih.gov/sra/?term=PRJNA915202).

## Supplementary material

SUPPLEMENTARY FIGURE S1 An IGV snapshot showing the BCR-ABL1 fusion transcript with reads from the alignment file mapping to the junction.

SUPPLEMENTARY FIGURE S2
**(A)** ORF sequence predicted by the orf maker using the fusion transcript, along with the amino acid sequence of the orf. The amino acid sequence shows similarity with arf-GAP GTPase domain on performing a protein blast. **(B)** The orfs from the partner genes in the fusion transcript HOXD11 and AGAP3 along with their protein domains.

SUPPLEMENTARY FIGURE S3 Boxplot showing the difference in TTYH expression between control and AML samples and a Kaplan Meier plot showing significant difference in survival probabilities between low and high TTYH3 expression.

SUPPLEMENTARY FIGURE S4 A heatmap of lncRNA-mRNA pairs with upregulated expression profiles specific to the HOXD11-AGAP3 family of patients.

SUPPLEMENTARY FIGURE S5 Bar chart showing significantly enriched pathways obtained using the genes of the “black” module correlating to the BCR group of samples.

SUPPLEMENTARY FIGURE S6 CCL28 and DOCK2 expression patterns across all the AML patients with upregulation specific to the HOXD11-AGAP3 category of patients along with a Kapalan-Meier curve of CCL28 in AML from GEPIA between low and high CCL28 expression groups.

SUPPLEMENTARY TABLE S1 Number of reads mapping to the Fusion Junctions in respective samples.
