# Supplementary material for: Whole transcriptome sequencing reveals HOXD11-AGAP3, a novel fusion transcript in the Indian acute leukemia cohort
Source: Front Genet. 2023 Apr 11;14:1100587. doi: 10.3389/fgene.2023.1100587 (PMC10126405; doi:10.3389/fgene.2023.1100587)
Supplement: Supplementary file 1 [file Presentation1.pptx]

## Slide 1
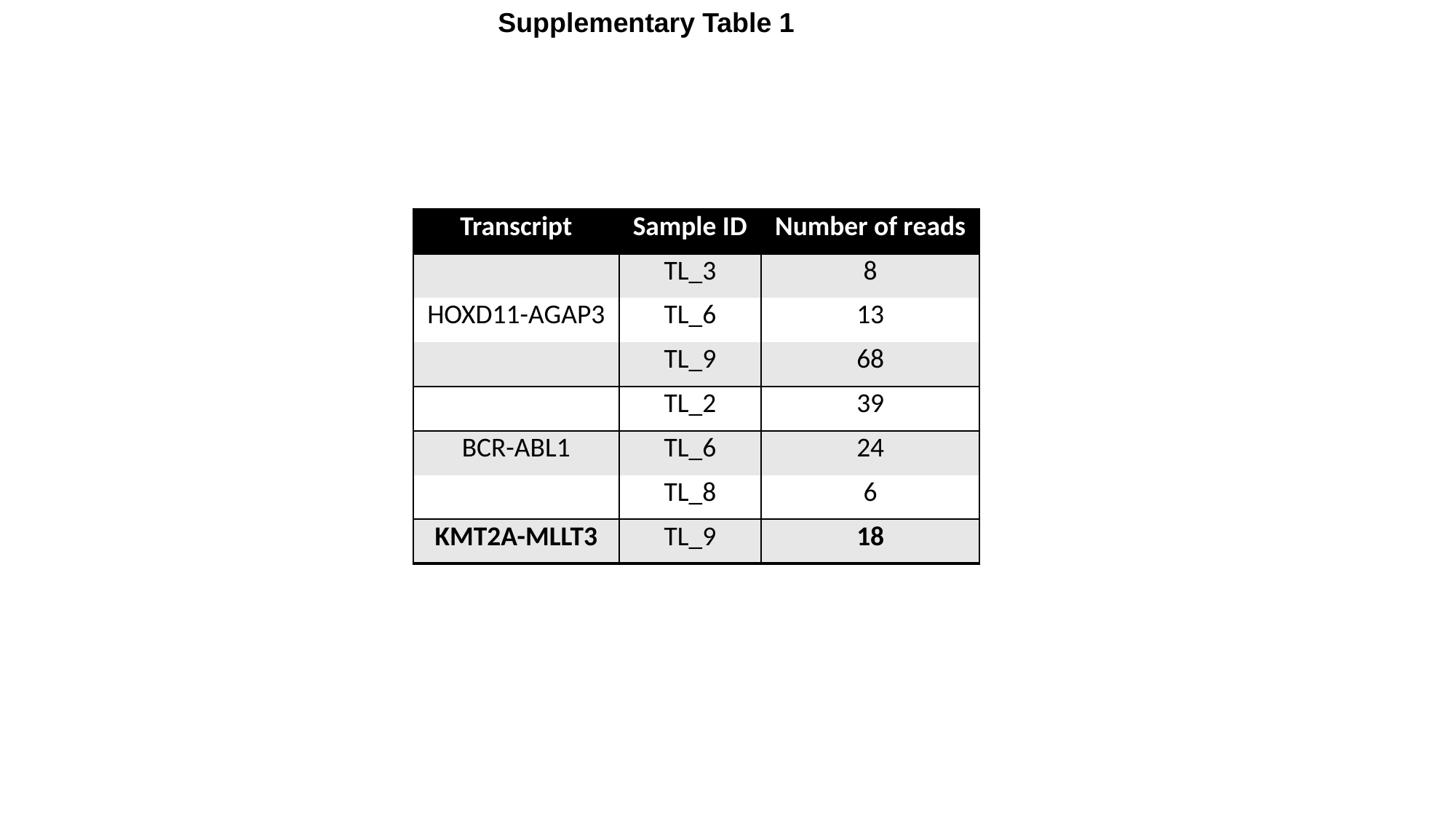

Supplementary Table 1
| Transcript | Sample ID | Number of reads |
| --- | --- | --- |
| | TL\_3 | 8 |
| HOXD11-AGAP3 | TL\_6 | 13 |
| | TL\_9 | 68 |
| | TL\_2 | 39 |
| BCR-ABL1 | TL\_6 | 24 |
| | TL\_8 | 6 |
| KMT2A-MLLT3 | TL\_9 | 18 |

## Slide 2
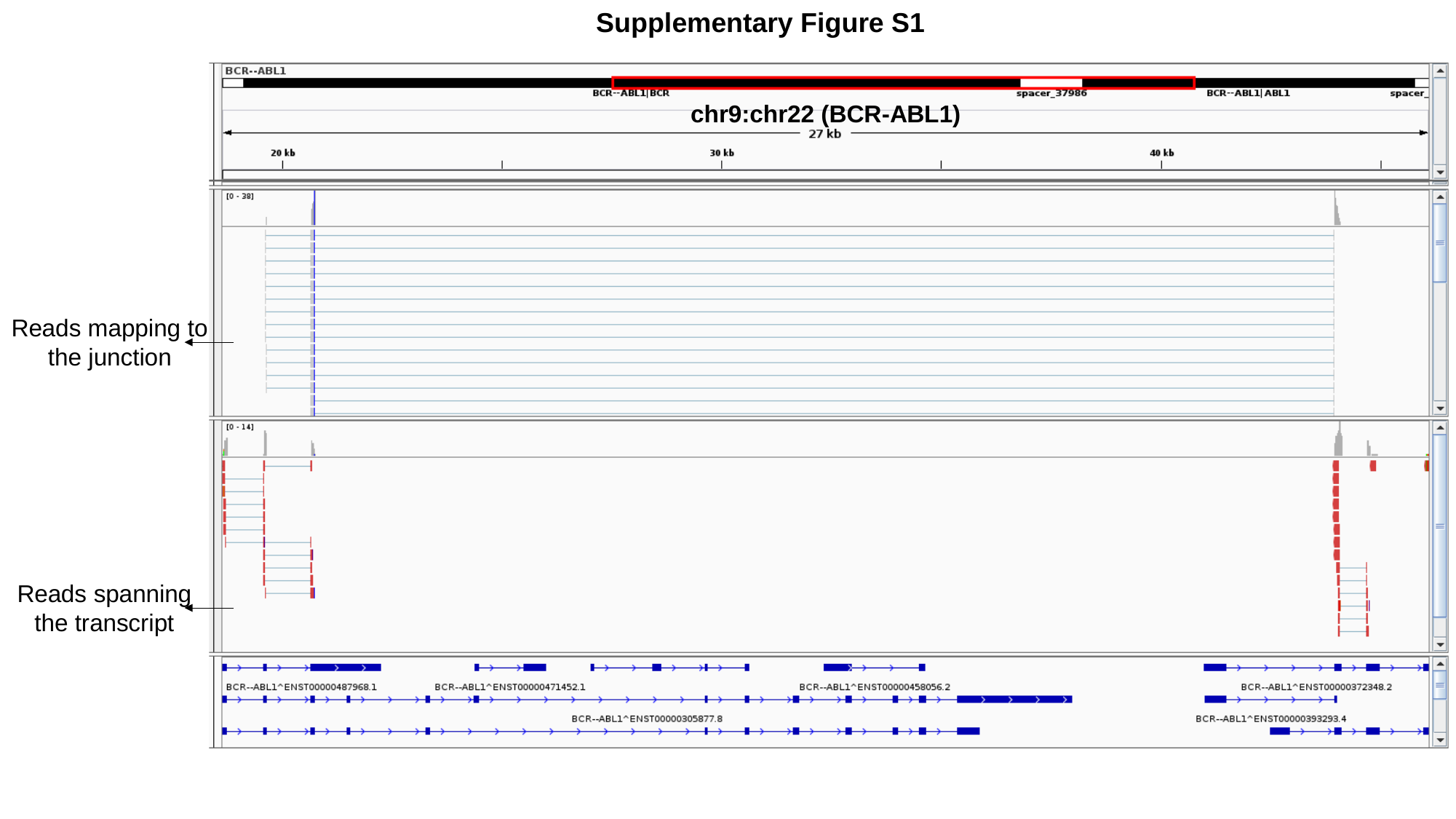

Supplementary Figure S1

## Slide 3
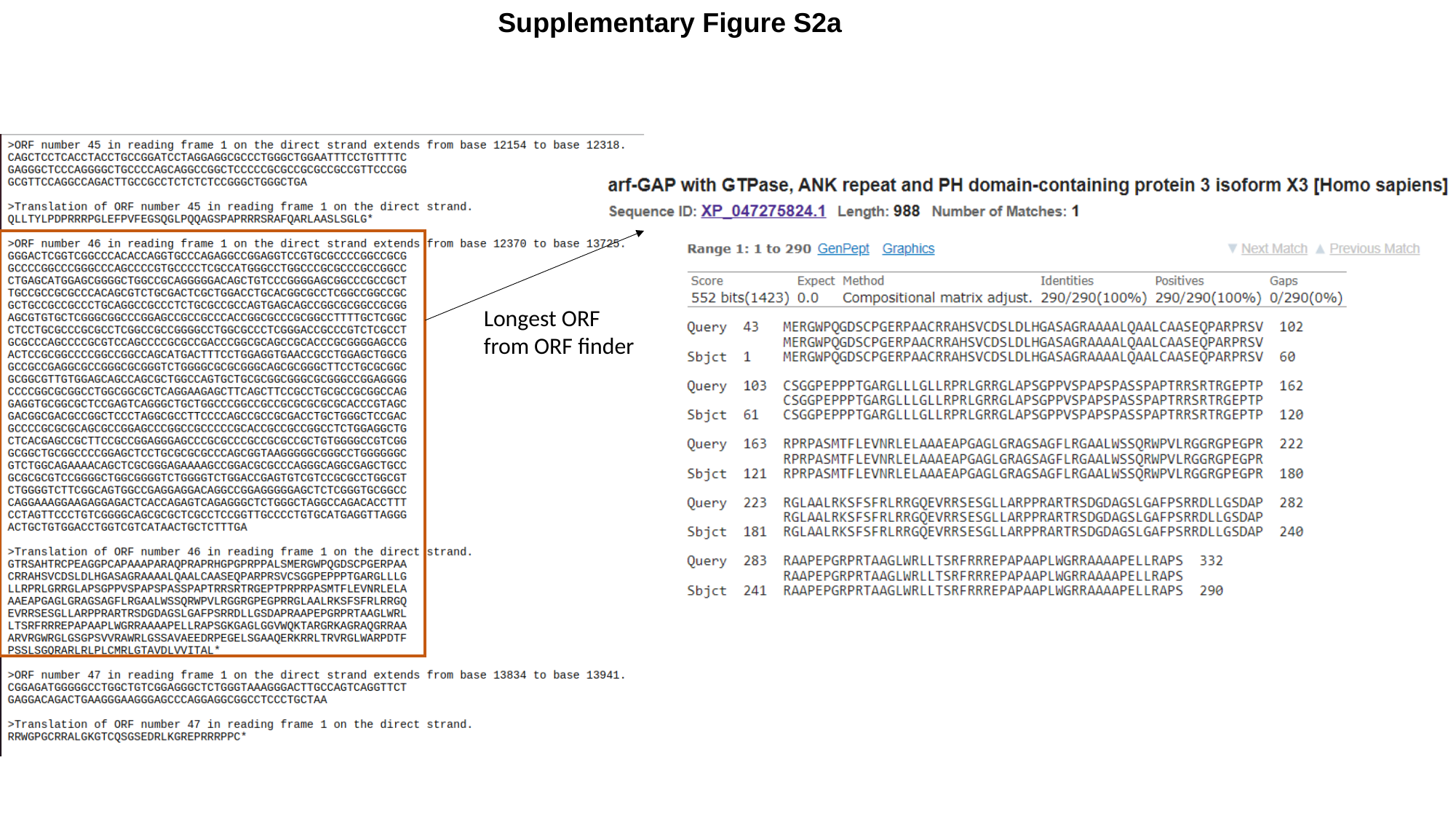

Supplementary Figure S2a

## Slide 4
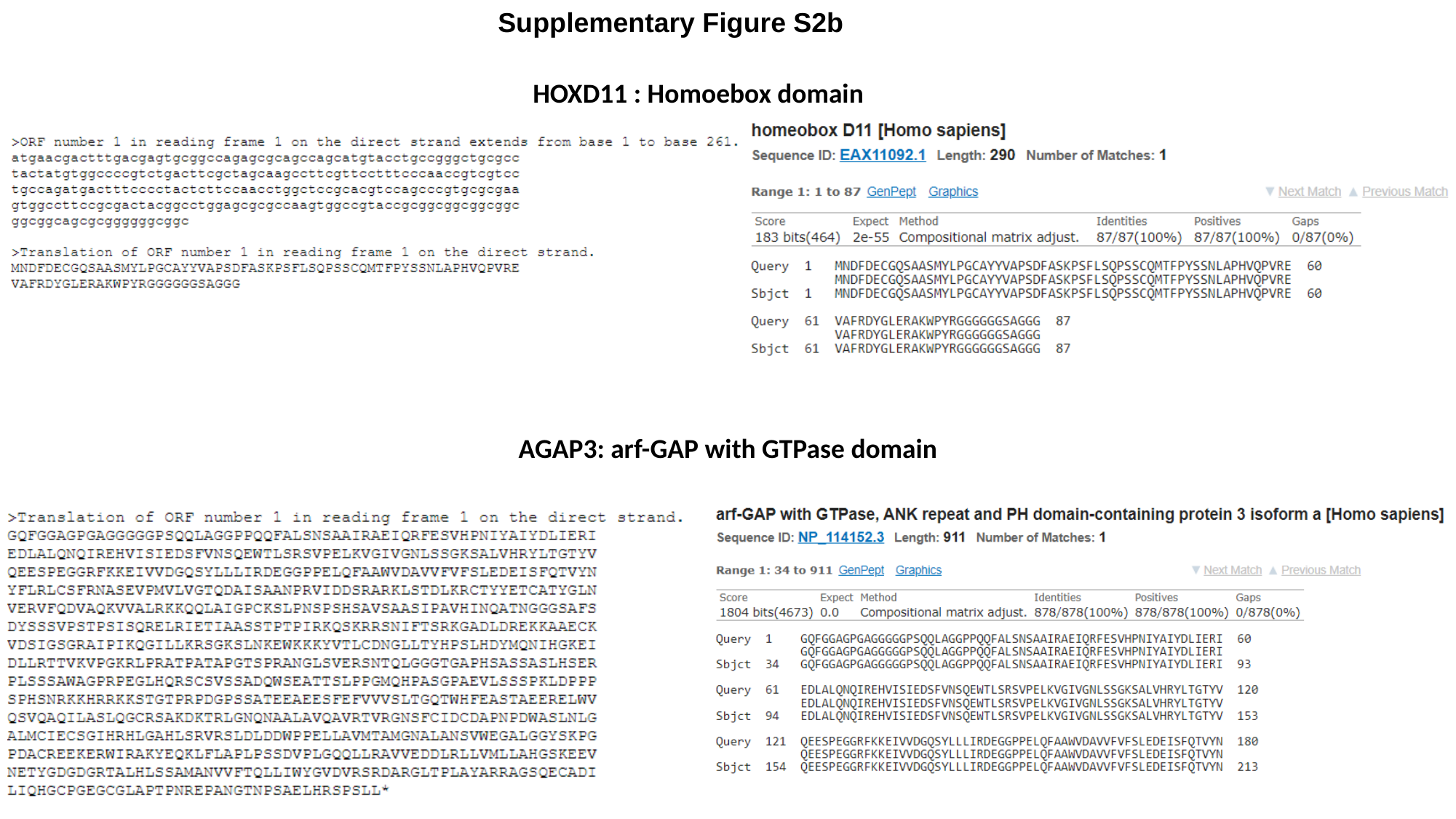

Supplementary Figure S2b
HOXD11 : Homoebox domain
AGAP3: arf-GAP with GTPase domain

## Slide 5
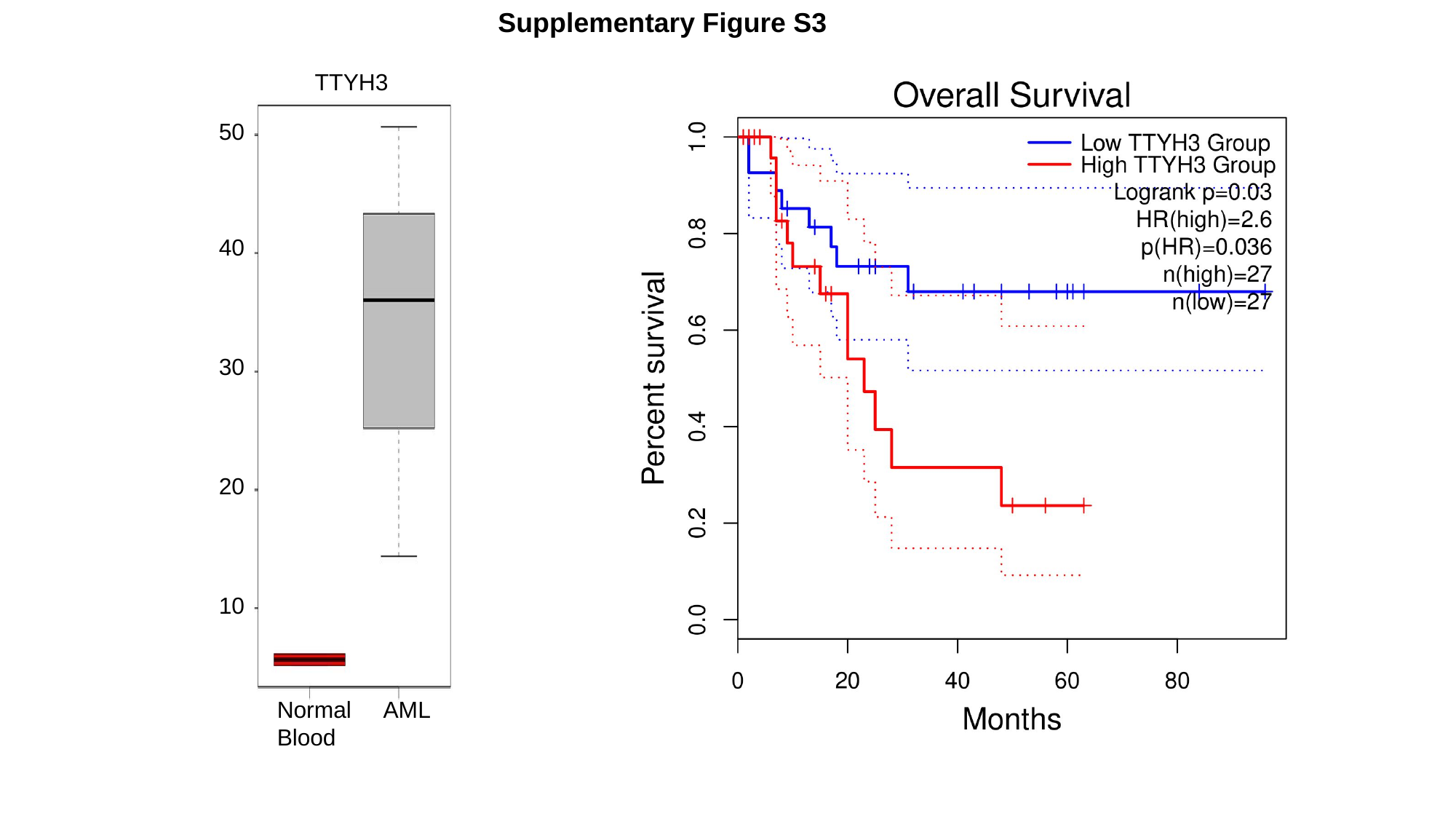

Supplementary Figure S3

## Slide 6
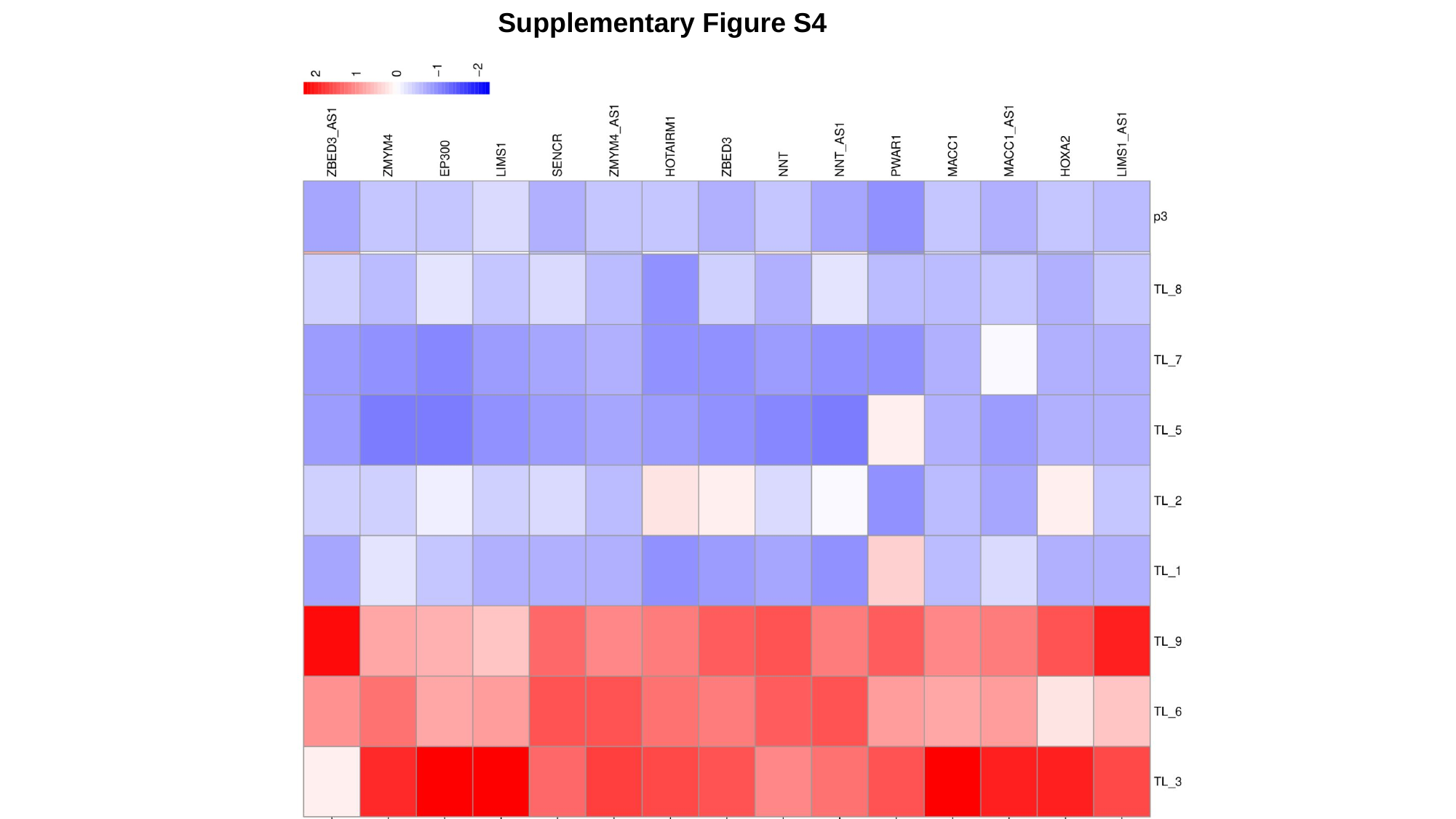

Supplementary Figure S4

## Slide 7
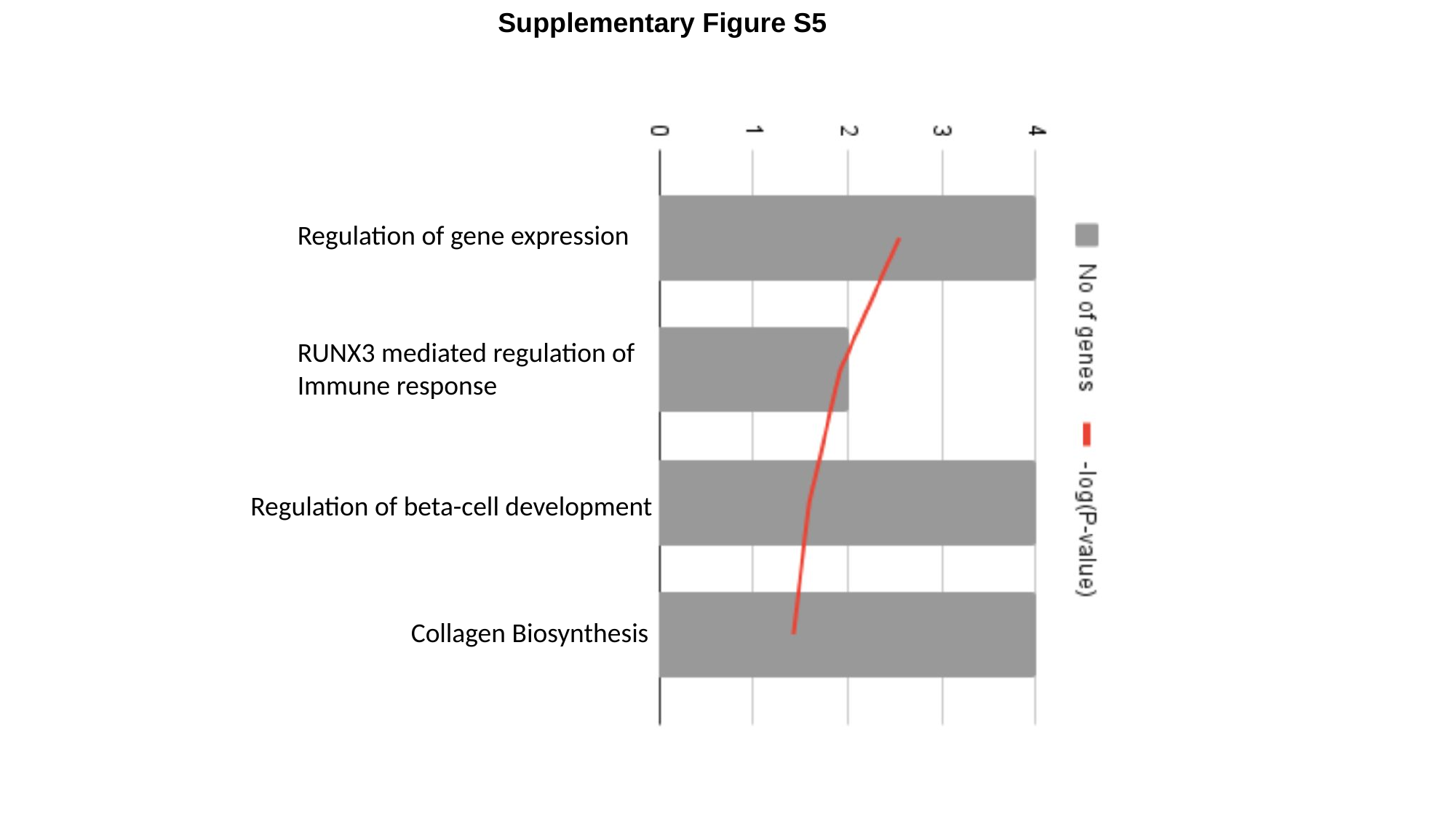

Supplementary Figure S5
Regulation of gene expression
RUNX3 mediated regulation of Immune response
Regulation of beta-cell development
Collagen Biosynthesis

## Slide 8
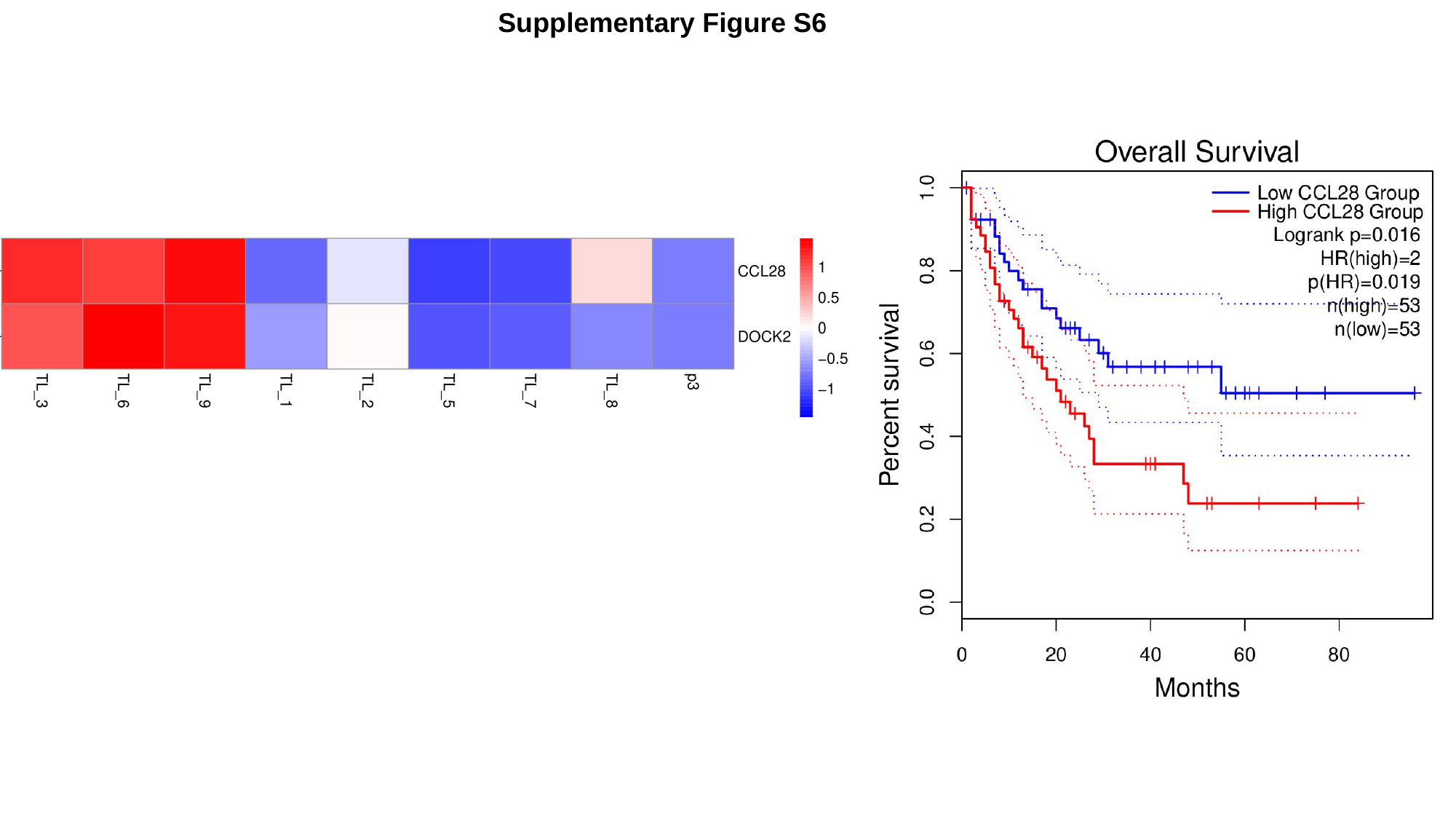

Supplementary Figure S6
