# Supplementary material for: Whole transcriptome sequencing reveals HOXD11-AGAP3, a novel fusion transcript in the Indian acute leukemia cohort
Source: Front Genet. 2023 Apr 11;14:1100587. doi: 10.3389/fgene.2023.1100587 (PMC10126405; doi:10.3389/fgene.2023.1100587)
Supplement: Supplementary file 2 [file DataSheet1.docx]

Supplementary Material

TL_3 Fusion Sequence:

**atgaacgactttgacgagtgcggccagagcgcagccagcatgtacctgccgggctgcgcctactatgtggccccgtctgacttcgctagcaagccttcgttcctttcccaaccgtcgtcctgccagatgactttcccctactcttccaacctggctccgcacgtccagcccgtgcgcgaagtggccttccgcgactacggcctggagcgcgccaagtggccgtaccgcggcggcggcggcggcggcagcgcggggggcggc|GGGCAGTTCGGCGGCGCGGGGCCCGGGGCCGGGGGCGGCGGCGGCCCCTCGCAGCAGCTGGCCGGCGGGCCCCCCCAGCAGTTCGCGCTCTCCAACTCCGCGGCCATCCGGGCCGAGATCCAGCGCTTCGAGTCCGTGCATCCCAATATCTACGCCATCTACGACCTGATCGAGCGCATCGAGGATTTGGCGCTGCAGAACCAGATCCGGGAGCACGTCATCTCCATCGAGGACTCGTTTGTGAACAGCCAGGAGTGGACGCTGAGCCGCTCCGTACCGGAGCTTAAAGTGGGCATAGTGGGGAACCTGTCTAGCGGGAAGTCAGCCCTGGTGCACCGCTATCTGACGGGGACCTATGTCCAGGAGGAGTCCCCTGAAGGGGGGCGGTTTAAGAAGGAGATTGTGGTGGATGGCCAGAGTTACCTGCTGCTGATCCGAGATGAAGGAGGCCCCCCTGAGCTCCAGTTTGCTGCCTGGGTGGATGCAGTGGTGTTTGTGTTCAGCCTGGAGGATGAAATCAGTTTCCAGACGGTGTACAACTACTTCCTGCGTCTCTGCAGCTTCCGCAACGCCAGCGAGGTGCCCATGGTGCTTGTGGGCACGCAGGATGCCATCAGCGCTGCGAATCCCCGGGTTATCGACGACAGCAGAGCCCGCAAGCTCTCCACAGATCTGAAGCGGTGCACCTACTATGAGACGTGCGCGACCTACGGGCTCAATGTGGAGCGTGTCTTCCAGGACGTGGCCCAGAAGGTAGTGGCCTTGCGAAAGAAGCAGCAACTGGCCATCGGGCCCTGCAAGTCACTGCCCAACTCGCCCAGCCACTCGGCCGTGTCCGCCGCCTCCATCCCGGCCGTGCACATCAACCAGGCCACGAATGGCGGCGGCAGCGCCTTCAGCGACTACTCGTCCTCAGTCCCCTCCACCCCCAGCATCAGCCAGCGGGAGCTGCGCATCGAGACCATCGCTGCCTCCTCCACCCCCACACCCATCCGAAAGCAGTCCAAGCGGCGCTCCAACATCTTCACGTCTCGGAAGGGTGCTGACCTGGACCGGGAGAAGAAGGCTGCCGAGTGCAAGGTGGACAGCATCGGGAGCGGCCGCGCCATCCCCATCAAGCAGGGGATCCTGCTAAAGCGGAGCGGCAAGTCCCTGAACAAGGAGTGGAAGAAGAAGTATGTGACGCTCTGTGACAACGGGCTGCTCACCTATCACCCCAGCCTGCATGATTACATGCAGAACATCCACGGCAAGGAGATTGACCTGCTGCGGACAACGGTGAAAGTGCCAGGGAAGCGCCTGCCCCGAGCCACACCTGCCACAGCCCCGGGCACCAGCCCCCGTGCCAACGGGCTGTCCGTGGAGCGGAGTAACACACAGCTGGGTGGGGGCACAGGTGCCCCCCACTCGGCCAGCAGCGCATCCCTGCACTCTGAGCGCCCCCTCAGCAGCTCGGCCTGGGCTGGCCCGCGCCCTGAGGGGCTGCACCAGCGCTCCTGCTCCGTTTCCAGCGCCGACCAGTGGAGTGAGGCCACCACTTCCCTGCCCCCAGGCATGCAGCACCCTGCCAGTGGCCCAGCTGAGGTACTCAGTTCCAGCCCCAAGCTGGATCCTCCCCCATCTCCCCACTCCAACCGGAAGAAGCACCGGAGGAAAAAGAGCACCGGGACCCCCCGACCAGACGGCCCCAGCAGTGCTACTGAAGAGGCAGAGGAGTCGTTTGAATTTGTGGTGGTGTCCCTCACTGGGCAGACGTGGCACTTCGAGGCTTCAACGGCGGAGGAGCGGGAGCTGTGGGTTCAGAGTGTGCAGGCCCAGATCCTTGCCAGCCTGCAAGGCTGCCGCAGTGCCAAGGACAAGACTCGACTGGGGAACCAGAACGCAGCTCTGGCTGTGCAGGCCGTCCGCACCGTCCGCGGCAACAGCTTTTGTATCGACTGCGATGCACCCAATCCAGACTGGGCCAGCCTGAACCTGGGTGCCCTGATGTGCATTGAGTGCTCAGGCATCCACCGACACCTGGGGGCTCACCTGTCCCGGGTGCGCTCCCTTGACCTCGATGACTGGCCGCCTGAGCTGCTGGCTGTCATGACTGCCATGGGCAATGCCCTCGCCAACAGCGTCTGGGAGGGGGCCTTGGGTGGCTACTCCAAGCCAGGGCCTGATGCCTGCAGAGAGGAGAAGGAACGCTGGATACGGGCCAAGTATGAACAGAAGCTCTTCCTGGCCCCACTGCCAAGCTCAGATGTGCCACTGGGGCAGCAGCTGCTCCGGGCCGTGGTGGAAGATGACCTGCGGCTGTTGGTGATGCTCCTGGCACATGGCTCCAAAGAGGAGGTGAATGAGACCTATGGGGACGGGGACGGGCGGACGGCTCTACATCTCTCCAGTGCCATGGCCAACGTTGTCTTCACGCAGCTGCTCATCTGGTACGGGGTGGACGTGAGGAGCCGGGACGCCCGGGGCCTGACTCCACTGGCATATGCTCGCCGGGCCGGCAGCCAGGAGTGTGCAGACATCTTGATCCAGCATGGCTGCCCTGGGGAGGGCTGTGGCTTAGCGCCTACCCCCAACAGAGAGCCTGCCAATGGCACCAACCCCTCTGCTGAGCTGCACCGTAGTCCTAGCCTCCTATAA**

TL_6 Fusion Sequence:

**atgaacgactttgacgagtgcggccagagcgcagccagcatgtacctgccgggctgcgcctactatgtggccccgtctgacttcgctagcaagccttcgttcctttcccaaccgtcgtcctgccagatgactttcccctactcttccaacctggctccgcacgtccagcccgtgcgcgaagtggccttccgcgactacggcctggagcgcgccaagtggccgtaccgcggcggcggcggcggcggcagcgcggggggcggc|GGGCAGTTCGGCGGCGCGGGGCCCGGGGCCGGGGGCGGCGGCGGCCCCTCGCAGCAGCTGGCCGGCGGGCCCCCCCAGCAGTTCGCGCTCTCCAACTCCGCGGCCATCCGGGCCGAGATCCAGCGCTTCGAGTCCGTGCATCCCAATATCTACGCCATCTACGACCTGATCGAGCGCATCGAGGATTTGGCGCTGCAGAACCAGATCCGGGAGCACGTCATCTCCATCGAGGACTCGTTTGTGAACAGCCAGGAGTGGACGCTGAGCCGCTCCGTACCGGAGCTTAAAGTGGGCATAGTGGGGAACCTGTCTAGCGGGAAGTCAGCCCTGGTGCACCGCTATCTGACGGGGACCTATGTCCAGGAGGAGTCCCCTGAAGGGGGGCGGTTTAAGAAGGAGATTGTGGTGGATGGCCAGAGTTACCTGCTGCTGATCCGAGATGAAGGAGGCCCCCCTGAGCTCCAGTTTGCTGCCTGGGTGGATGCAGTGGTGTTTGTGTTCAGCCTGGAGGATGAAATCAGTTTCCAGACGGTGTACAACTACTTCCTGCGTCTCTGCAGCTTCCGCAACGCCAGCGAGGTGCCCATGGTGCTTGTGGGCACGCAGGATGCCATCAGCGCTGCGAATCCCCGGGTTATCGACGACAGCAGAGCCCGCAAGCTCTCCACAGATCTGAAGCGGTGCACCTACTATGAGACGTGCGCGACCTACGGGCTCAATGTGGAGCGTGTCTTCCAGGACGTGGCCCAGAAGGTAGTGGCCTTGCGAAAGAAGCAGCAACTGGCCATCGGGCCCTGCAAGTCACTGCCCAACTCGCCCAGCCACTCGGCCGTGTCCGCCGCCTCCATCCCGGCCGTGCACATCAACCAGGCCACGAATGGCGGCGGCAGCGCCTTCAGCGACTACTCGTCCTCAGTCCCCTCCACCCCCAGCATCAGCCAGCGGGAGCTGCGCATCGAGACCATCGCTGCCTCCTCCACCCCCACACCCATCCGAAAGCAGTCCAAGCGGCGCTCCAACATCTTCACGTCTCGGAAGGGTGCTGACCTGGACCGGGAGAAGAAGGCTGCCGAGTGCAAGGTGGACAGCATCGGGAGCGGCCGCGCCATCCCCATCAAGCAGGGGATCCTGCTAAAGCGGAGCGGCAAGTCCCTGAACAAGGAGTGGAAGAAGAAGTATGTGACGCTCTGTGACAACGGGCTGCTCACCTATCACCCCAGCCTGCATGATTACATGCAGAACATCCACGGCAAGGAGATTGACCTGCTGCGGACAACGGTGAAAGTGCCAGGGAAGCGCCTGCCCCGAGCCACACCTGCCACAGCCCCGGGCACCAGCCCCCGTGCCAACGGGCTGTCCGTGGAGCGGAGTAACACACAGCTGGGTGGGGGCACAGGTGCCCCCCACTCGGCCAGCAGCGCATCCCTGCACTCTGAGCGCCCCCTCAGCAGCTCGGCCTGGGCTGGCCCGCGCCCTGAGGGGCTGCACCAGCGCTCCTGCTCCGTTTCCAGCGCCGACCAGTGGAGTGAGGCCACCACTTCCCTGCCCCCAGGCATGCAGCACCCTGCCAGTGGCCCAGCTGAGGTACTCAGTTCCAGCCCCAAGCTGGATCCTCCCCCATCTCCCCACTCCAACCGGAAGAAGCACCGGAGGAAAAAGAGCACCGGGACCCCCCGACCAGACGGCCCCAGCAGTGCTACTGAAGAGGCAGAGGAGTCGTTTGAATTTGTGGTGGTGTCCCTCACTGGGCAGACGTGGCACTTCGAGGCTTCAACGGCGGAGGAGCGGGAGCTGTGGGTTCAGAGTGTGCAGGCCCAGATCCTTGCCAGCCTGCAAGGCTGCCGCAGTGCCAAGGACAAGACTCGACTGGGGAACCAGAACGCAGCTCTGGCTGTGCAGGCCGTCCGCACCGTCCGCGGCAACAGCTTTTGTATCGACTGCGATGCACCCAATCCAGACTGGGCCAGCCTGAACCTGGGTGCCCTGATGTGCATTGAGTGCTCAGGCATCCACCGACACCTGGGGGCTCACCTGTCCCGGGTGCGCTCCCTTGACCTCGATGACTGGCCGCCTGAGCTGCTGGCTGTCATGACTGCCATGGGCAATGCCCTCGCCAACAGCGTCTGGGAGGGGGCCTTGGGTGGCTACTCCAAGCCAGGGCCTGATGCCTGCAGAGAGGAGAAGGAACGCTGGATACGGGCCAAGTATGAACAGAAGCTCTTCCTGGCCCCACTGCCAAGCTCAGATGTGCCACTGGGGCAGCAGCTGCTCCGGGCCGTGGTGGAAGATGACCTGCGGCTGTTGGTGATGCTCCTGGCACATGGCTCCAAAGAGGAGGTGAATGAGACCTATGGGGACGGGGACGGGCGGACGGCTCTACATCTCTCCAGTGCCATGGCCAACGTTGTCTTCACGCAGCTGCTCATCTGGTACGGGGTGGACGTGAGGAGCCGGGACGCCCGGGGCCTGACTCCACTGGCATATGCTCGCCGGGCCGGCAGCCAGGAGTGTGCAGACATCTTGATCCAGCATGGCTGCCCTGGGGAGGGCTGTGGCTTAGCGCCTACCCCCAACAGAGAGCCTGCCAATGGCACCAACCCCTCTGCTGAGCTGCACCGTAGTCCTAGCCTCCTATAA**

TL_9 Fusion Sequence:

**atgaacgactttgacgagtgcggccagagcgcagccagcatgtacctgccgggctgcgcctactatgtggccccgtctgacttcgctagcaagccttcgttcctttcccaaccgtcgtcctgccagatgactttcccctactcttccaacctggctccgcacgtccagcccgtgcgcgaagtggccttccgcgactacggcctggagcgcgccaagtggccgtaccgcggcggcggcggcggcggcagcgcggggggcggc|GGGCAGTTCGGCGGCGCGGGGCCCGGGGCCGGGGGCGGCGGCGGCCCCTCGCAGCAGCTGGCCGGCGGGCCCCCCCAGCAGTTCGCGCTCTCCAACTCCGCGGCCATCCGGGCCGAGATCCAGCGCTTCGAGTCCGTGCATCCCAATATCTACGCCATCTACGACCTGATCGAGCGCATCGAGGATTTGGCGCTGCAGAACCAGATCCGGGAGCACGTCATCTCCATCGAGGACTCGTTTGTGAACAGCCAGGAGTGGACGCTGAGCCGCTCCGTACCGGAGCTTAAAGTGGGCATAGTGGGGAACCTGTCTAGCGGGAAGTCAGCCCTGGTGCACCGCTATCTGACGGGGACCTATGTCCAGGAGGAGTCCCCTGAAGGGGGGCGGTTTAAGAAGGAGATTGTGGTGGATGGCCAGAGTTACCTGCTGCTGATCCGAGATGAAGGAGGCCCCCCTGAGCTCCAGTTTGCTGCCTGGGTGGATGCAGTGGTGTTTGTGTTCAGCCTGGAGGATGAAATCAGTTTCCAGACGGTGTACAACTACTTCCTGCGTCTCTGCAGCTTCCGCAACGCCAGCGAGGTGCCCATGGTGCTTGTGGGCACGCAGGATGCCATCAGCGCTGCGAATCCCCGGGTTATCGACGACAGCAGAGCCCGCAAGCTCTCCACAGATCTGAAGCGGTGCACCTACTATGAGACGTGCGCGACCTACGGGCTCAATGTGGAGCGTGTCTTCCAGGACGTGGCCCAGAAGGTAGTGGCCTTGCGAAAGAAGCAGCAACTGGCCATCGGGCCCTGCAAGTCACTGCCCAACTCGCCCAGCCACTCGGCCGTGTCCGCCGCCTCCATCCCGGCCGTGCACATCAACCAGGCCACGAATGGCGGCGGCAGCGCCTTCAGCGACTACTCGTCCTCAGTCCCCTCCACCCCCAGCATCAGCCAGCGGGAGCTGCGCATCGAGACCATCGCTGCCTCCTCCACCCCCACACCCATCCGAAAGCAGTCCAAGCGGCGCTCCAACATCTTCACGTCTCGGAAGGGTGCTGACCTGGACCGGGAGAAGAAGGCTGCCGAGTGCAAGGTGGACAGCATCGGGAGCGGCCGCGCCATCCCCATCAAGCAGGGGATCCTGCTAAAGCGGAGCGGCAAGTCCCTGAACAAGGAGTGGAAGAAGAAGTATGTGACGCTCTGTGACAACGGGCTGCTCACCTATCACCCCAGCCTGCATGATTACATGCAGAACATCCACGGCAAGGAGATTGACCTGCTGCGGACAACGGTGAAAGTGCCAGGGAAGCGCCTGCCCCGAGCCACACCTGCCACAGCCCCGGGCACCAGCCCCCGTGCCAACGGGCTGTCCGTGGAGCGGAGTAACACACAGCTGGGTGGGGGCACAGGTGCCCCCCACTCGGCCAGCAGCGCATCCCTGCACTCTGAGCGCCCCCTCAGCAGCTCGGCCTGGGCTGGCCCGCGCCCTGAGGGGCTGCACCAGCGCTCCTGCTCCGTTTCCAGCGCCGACCAGTGGAGTGAGGCCACCACTTCCCTGCCCCCAGGCATGCAGCACCCTGCCAGTGGCCCAGCTGAGGTACTCAGTTCCAGCCCCAAGCTGGATCCTCCCCCATCTCCCCACTCCAACCGGAAGAAGCACCGGAGGAAAAAGAGCACCGGGACCCCCCGACCAGACGGCCCCAGCAGTGCTACTGAAGAGGCAGAGGAGTCGTTTGAATTTGTGGTGGTGTCCCTCACTGGGCAGACGTGGCACTTCGAGGCTTCAACGGCGGAGGAGCGGGAGCTGTGGGTTCAGAGTGTGCAGGCCCAGATCCTTGCCAGCCTGCAAGGCTGCCGCAGTGCCAAGGACAAGACTCGACTGGGGAACCAGAACGCAGCTCTGGCTGTGCAGGCCGTCCGCACCGTCCGCGGCAACAGCTTTTGTATCGACTGCGATGCACCCAATCCAGACTGGGCCAGCCTGAACCTGGGTGCCCTGATGTGCATTGAGTGCTCAGGCATCCACCGACACCTGGGGGCTCACCTGTCCCGGGTGCGCTCCCTTGACCTCGATGACTGGCCGCCTGAGCTGCTGGCTGTCATGACTGCCATGGGCAATGCCCTCGCCAACAGCGTCTGGGAGGGGGCCTTGGGTGGCTACTCCAAGCCAGGGCCTGATGCCTGCAGAGAGGAGAAGGAACGCTGGATACGGGCCAAGTATGAACAGAAGCTCTTCCTGGCCCCACTGCCAAGCTCAGATGTGCCACTGGGGCAGCAGCTGCTCCGGGCCGTGGTGGAAGATGACCTGCGGCTGTTGGTGATGCTCCTGGCACATGGCTCCAAAGAGGAGGTGAATGAGACCTATGGGGACGGGGACGGGCGGACGGCTCTACATCTCTCCAGTGCCATGGCCAACGTTGTCTTCACGCAGCTGCTCATCTGGTACGGGGTGGACGTGAGGAGCCGGGACGCCCGGGGCCTGACTCCACTGGCATATGCTCGCCGGGCCGGCAGCCAGGAGTGTGCAGACATCTTGATCCAGCATGGCTGCCCTGGGGAGGGCTGTGGCTTAGCGCCTACCCCCAACAGAGAGCCTGCCAATGGCACCAACCCCTCTGCTGAGCTGCACCGTAGTCCTAGCCTCCTATAA**
